# Polypharmacy, potentially inappropriate medications and associated factors among older adults with hypertension in primary care

**DOI:** 10.1590/0034-7167-2022-0785

**Published:** 2023-12-08

**Authors:** Carine Teles Sangaleti, Maicon Henrique Lentsck, Dannyele Cristina da Silva, Andrielli Machado, Maria Regiane Trincaus, Maria Cristina Umpierrez Vieira, Bruno Bordin Pelazza, Fernanda Marciano Consolim Colombo

**Affiliations:** I Universidade Estadual do Centro-Oeste. Guarapuava, Paraná, Brazil; II Hospital São Vicente de Paula. Guarapuava, Paraná, Brazil; III Universidade de São Paulo, Faculdade de Medicina, Instituto do Coração, Unidade de Hipertensão. São Paulo, São Paulo, Brazil

**Keywords:** Hypertension, Aged, Polypharmacy, Potentially Inappropriate Medication List, Primary Care, Hipertensión, Anciano, Polifarmacia, Lista de Medicamentos Potencialmente Inapropiados, Atención Primaria, Hipertensão, Idoso, Polifarmácia, Medicamentos Potencialmente Inapropriados, Atenção Básica

## Abstract

**Objective:**

to identify the prevalence and associations of polypharmacy and potentially inappropriate medication use among older adults with hypertension treated in primary care.

**Methods:**

a cross-sectional study carried out with older adults with hypertension treated at a Family Health Strategy unit. Data collection included analysis of medical records, interviews and multidimensional assessment of older adults. Socio-demographic information and clinical variables were collected. Statistical analysis was performed by multiple logistic regression.

**Results:**

polypharmacy prevalence was 38.09%, and potentially inappropriate medication (PIM), 28.57%. There was a significant association between polypharmacy and PIM use, altered sleep and ethnicity. PIM use was associated with polypharmacy, worse family functioning, and absence of a caregiver. Cognitive decline reduces the prevalence of these medications.

**Conclusions:**

polypharmacy and PIM use among older adults with hypertension represent a problem in this population, especially among the most vulnerable.

## RESUMO

**Objetivo:**

identificar a prevalência e as associações da polifarmácia e uso de medicamentos potencialmente inapropriados entre idosos com hipertensão atendidos na atenção básica.

**Métodos:**

estudo transversal, realizado com idosos com hipertensão atendidos em unidade da Estratégia Saúde da Família. A coleta de dados englobou análise de prontuário, entrevista e avaliação multidimensional dos idosos. Foram coletadas informações socioedemográficas e variáveis clínicas. A análise estatística se deu por regressão logística múltipla.

**Resultados:**

a prevalência de polifarmácia foi de 38,09%, e de medicamentos potencialmente inapropriados (MPI), de 28,57%. Houve associação significativa entre polifarmácia e uso de MPI, sono alterado e etnia. O uso de MPI foi associado à polifarmácia, pior funcionalidade familiar e ausência de cuidador. O declínio cognitivo reduz a prevalência destes medicamentos.

**Conclusões:**

a polifarmácia e o uso de MPI entre idosos com hipertensão representam um problema nessa população, especialmente entre aqueles mais vulneráveis.

## RESUMEN

**Objetivo:**

identificar la prevalencia y asociaciones de polifarmacia y uso de medicamentos potencialmente inapropiados entre ancianos hipertensos atendidos en atención primaria.

**Métodos:**

estudio transversal, realizado con ancianos hipertensos atendidos en una unidad de la Estrategia Salud de la Familia. La recolección de datos incluyó análisis de historias clínicas, entrevistas y evaluación multidimensional de los ancianos. Se recogió información sociodemográfica y variables clínicas. El análisis estadístico se realizó mediante regresión logística múltiple.

**Resultados:**

la prevalencia de polifarmacia fue de 38,09% y de medicación potencialmente inapropiada (MPI), de 28,57%. Hubo una asociación significativa entre la polifarmacia y el uso de MPI, la alteración del sueño y el origen étnico. El uso de MPI se asoció con polifarmacia, peor funcionamiento familiar y ausencia de un cuidador. El deterioro cognitivo reduce la prevalencia de estos medicamentos.

**Conclusiones:**

la polifarmacia y el uso de MPI entre personas mayores con hipertensión representan un problema en esta población, especialmente entre los más vulnerables.

## INTRODUCTION

It is well established that effective treatment of hypertension reduces morbidity and mortality resulting from this clinical condition^(1)^. Its control represents one of the greatest public health challenges, especially for primary care services of the Brazilian Health System (SUS – *Sistema Único de Saúde*), which are the main reference for hypertensive patients in our country. There is a need to adopt broad and more efficient care strategies in the work process of all members of primary care teams^(2)^, especially for the age groups most vulnerable to the deleterious effects of hypertension, such as the population over 60-65 years of age, in which the occurrence of problems such as adverse effects and polypharmacy can be detected^(3, 4)^.

According to data from the System for Surveillance of Risk and Protective Factors for Chronic Diseases by Telephone Survey (*Sistema de Vigilância de Fatores de Risco e Proteção para Doenças Crônicas por Inquérito Telefônico*)^(5)^, 60.9% of people aged over 65 years said they were hypertensive. When assessing older adults’ overall health, there is a high presence of target organ damage associated with hypertension as well as other risk factors such as Diabetes Mellitus, dyslipidemia, sleep, memory and mood disorders. Older adults can present with different degrees of fragility and, when very weak, depend on help from third parties for their personal care^(6)^. Thus, in addition to antihypertensive drugs, the management of various clinical alterations leads to multiple drug prescription, culminating in the condition called polypharmacy, which is the concomitant use of five or more medication items^(7)^.

Polypharmacy favors forgetting or duplicating doses and prescribing potentially inappropriate medications (PIM) for older adults^(8)^. PIM are those whose risks of adverse effects and negative outcomes outweigh their benefits and, therefore, should be avoided by older adults^(9)^.

It should be noted that negative outcomes associated with polypharmacy practice have been described, ranging from severe adverse drug reactions to increased mortality^(10)^. These outcomes are closely related to PIM prescription for older adults.

In this context, no less important is the inappropriate custom of self-medication, a condition encouraged by medicalization in old age. Also, a not well-structured care system leads older adults to go through different health services and, consequently, to receive several prescriptions, and they do not always consider an existing one^(11)^.

Brazil already has the sixth largest population of older adults on the planet; therefore, controlling hypertension and the co-morbidities that frequently affect this population, with special emphasis on different drug use, is a major public health challenge. It should be noted that there are few studies on polypharmacy and PIM use among older adults nationwide.

This study was guided by the hypothesis that individual characteristics, presence of other chronic diseases and clinical variables indicated for the multidimensional assessment of older adults are associated with polypharmacy and PIM. Therefore, analyzing the determinants of polypharmacy and PIM in older adults with hypertension in primary care becomes important to establish adequate and efficient care strategies capable of impacting primary care teams’ work process.

## OBJECTIVE

To identify the prevalence and associations of polypharmacy and PIM use among older adults with hypertension treated in primary care, with a view to improving hypertension and drug therapy management in this group.

## METHODS

### Ethical aspects

The research was approved by the Research Ethics Committee of the *Universidade Estadual do Centro-Oeste*, under the Certificate of Presentation for Ethical Consideration (CAAE - *Certificado de Apresentação para Apreciação Ética*). The Informed Consent Form was obtained from all those involved in the study in writing.

### Study design, period, and place

This is an analytical, observational, cross-sectional study, which was carried out in a Family Health Strategy unit, with an enrolled population of 4,525 people, in a medium-sized municipality in the Center-South region of the state of Paraná. The data presented reflect the scope of a broader study still in progress on the multidimensional assessment of older adults in primary care. The study design followed the checklist STrengthening the Reporting of OBservational studies in Epidemiology (STROBE) recommendations.

### Population and inclusion criteria

The target population consisted of older adults, aged 60 years or older, with hypertension and in regular care at the basic reference unit. Older adults with a diagnosis of hypertension in medical records, regular follow-up at the basic unit, who had at least one medical or nursing consultation and a visit from a community health worker within a year, were included.

The Family Health Strategy unit in which the study took place had 329 older adults registered; of these, 213 were classified as hypertensive, that is, 64.74% of the total number of older adults.

### Study protocol

Collection encompassed three sources of data: analysis of sociodemographic characteristics in medical records, record of other chronic conditions in addition to hypertension and record of prescribed medications; interview to confirm or collect sociodemographic data and to identify prescriptions that were not included in the medical record; and, to compose the analytical character of the study, a multidimensional assessment of all older adult participants was carried out according to SESA-PR^(12)^.

Multidimensional assessment was performed with all older adults eligible for the study. An invitation to participate in the study was made by community health workers or by active search by the collection team, in addition to referrals of older adults by health professionals at the unit. The next step was to schedule interviews and assessments in the basic unit with older adults.

Assessment took place at the health unit in a large room, used for holding educational groups, in order to allow functionality index assessment. Assessments lasted from 40 to 90 minutes. It should be noted that data collection took place only after explaining the study and reading and signing the Informed Consent Form.

If a participant could not complete the assessment on the scheduled day, a new appointment was established to complete it.

Data collection took place between July 2017 and July 2018, being conducted, in all its stages, by researchers with experience in the clinical assessment of older adults.

Classification of study variables and data collection procedures:

- Sociodemographic conditions: information collected from the medical record and interview, being: gender – male or female; self-declared race/color: white, black and brown (stratified as white and non-white); age in years; education classified according to years of study as: illiterate, incomplete elementary school, complete elementary school and incomplete high school, according to the municipality’s registration system; marital status classified as married, single, widowed, divorced and living together, according to the municipality’s registration system; income in number of minimum wages; number of children, type of housing that was classified as masonry and others; whether or not to rely on daily support from a home caregiver (family member or not);
- Concomitant presence of other chronic diseases: it was considered “yes” if older adults presented a record in the medical record, or informed in the interview that they had another chronic disease in addition to hypertension;
- Polypharmacy analysis and inappropriate medication prescription: from medical record, interview and prescription analysis. Older adults were classified in the polypharmacy regiment in simultaneous use of five or more drugs. And Beers-Fick^(12)^ criterion was used to classify PIM use;
- Clinical variables: assessed according to the parameters and instruments indicated for the multidimensional assessment of older adults in primary care^(13)^. The following instruments and parameters were used (Chart 1).

**Chart 1:** Parameters, instruments and classification adopted

Sleep quality	Mini Sleep Questionnaire (MSQ)	Adequate sleep: score 0-24 Altered sleep: score above 25
Cognitive assessment	Mini Mental State Examination (MMSE)	Normal – score equal to or greater than: 19.1 – illiterate older adults 23.4 – older adults with up to 3 years of education 24 – older adults with 3 to 7 years of education 28 – older adults with more than 7 years of education Altered - below the values mentioned above, considering the years of study
Depression	Geriatric Depression Scale (GDS)	Normal: score from 0-5 Altered: score from 6-15
Falls	Investigation of falls in the last 12 months	No – for no fall Yes – for older adults who fell within a 12-month period
Activity of Daily Living (ADL) assessment	Katz ADL Index	Normal – for older adults independent for all activities Altered: for older adults who have any need for support
Family functionality assessment	Family Apgar	0 to 4 = high family dysfunction 5 and 6 = moderate family dysfunction 7 to 10 = good family functionality
Fall risk assessment	Performance Oriented Mobility Assessment (POMA) score	Continuous variable scored from 22 (minimum) to 57 (maximum) The lower the score, the greater the risk of falls
Nutritional status assessment	Mini Nutritional Assessment (MAN)	The lower the value, the worse the nutritional status

### Analysis of results, and statistics

Data were analyzed descriptively, with central and dispersion measures, such as mean, standard deviation and median, and also through relative (%) and absolute (n) frequency. To compare categorical variables, Pearson’s chi-square test or Fisher’s exact test (for expected values less than 5) were performed. To compare numerical variables between two groups, Student’s t test or Mann-Whitney test were performed. The p-value≤0.05 was considered significant in each of the tests.

Multiple analysis was performed using multiple logistic regression models, using the stepwise forward model, which estimated the Odds Ratio (OR) and the respective confidence intervals (CI). Variables with p<0.20 in the univariate analysis were included in the model, and variables that remained significant (p<0.05) or that fitted the model were maintained in the final model. The adequacy of the final models was verified using the Deviance and Hosmer-Lemeshow tests. Variable collinearity was tested with variance inflation factor (VIF) and statistical analyzes performed with the Stata software version 12.

Moreover, the results were compared and analyzed according to the scientific literature relevant to the subject under study.

## RESULTS

A total of 189 older adults were assessed and included in this study; of these, 77.25% were between 60 and 79 years old and 22.75% were long-lived; 58.73% declared themselves white; 42.32% were married; 32.27% had no education; 41.27% had incomplete primary school; 39.15% lived in brick houses; 47.08% had 6 or more children; 83.60% had a family income of two to four minimum wages; and 85.18% have another chronic disease in addition to hypertension, with Diabetes Mellitus being the most frequent (24.84%).

The use of five or more medications was present among 38.09% of older adults, with 13 being the maximum number of medications found in simultaneous use. With regard to antihypertensive drugs, 30.15% of older adults were on antihypertensive monotherapy, mostly using angiotensin receptor blockers (ARBs); 22.22% used two antihypertensive medications; and 17.47% used three or more drugs of this class.

The prevalence of PIM prescription and continuous use for older adults was 28.57% (54 older adults), with proton pump inhibitors being the most frequent (20.10%), followed by antidepressants (14.28%), benzodiazepines (11.64%) and non-steroidal anti-inflammatory drugs (7.40%). Furthermore, 13.69% had a prescription and made continuous use of more than one medication contraindicated for older adults, and, among antihypertensive drugs, 6.87% of older adults used contraindicated classes. It should be noted that the prevalence of PIM use rises to 39.68% when considering recurrent, but not continuous, intake of these types of medication.

Table 1 presents the associations found between the use of five or more medications and sociodemographic variables among participants. It was identified that the presence of polypharmacy was associated with being female (p=0.017).

**Table 1:** Univariate analysis of factors associated with polypharmacy in older adults followed by the Family Health Strategy according to sociodemo-graphic profile. Guarapuava, Paraná, Brazil, 2022

Variables	Total	Polypharmacy				OR*	p value
		Yes		No			
	n	n	%	n	%		
Sex							0.017**
Male	70	19	26.4	51	43.6	Ref.	
Female	119	53	73.6	66	56.4	2.15	
Age group							0.622
60 to 79 years	146	57	79.2	89	76.1	Ref.	
80 years and older	43	15	20.8	28	23.9	0.83	
Race/color							0.056**
White	111	36	50.0	75	64.1	Ref.	
Non-white	78	36	50.0	42	35.9	1.78	
Marital status							0.357
Married	80	27	37.5	53	45.3	Ref.	
Singled	10	5	6.9	5	4.3	1.96	
Widowed	71	25	34.7	46	39.0	1.06	
Divorced	22	12	16.7	120	8.5	2.35	
Living together	6	3	4.2	3	2.6	1.96	
Number of children							0.132**
0 to 5	105	35	48.6	77	59.8	Ref.	
6 and more	84	37	51.4	47	40.2	1.57	
Income							0.256
1 wage	31	9	12.5	22	18.8	Ref.	
2 to 4 wages	158	63	87.5	95	81.2	1.62	
Education							0.563
Illiterate	61	24	33.3	37	31.6	1.62	
Incomplete elementary school	78	33	45.8	45	38.5	1.83	
Complete elementary school	29	9	12.5	20	17.1	1.12	
Incomplete high school	21	6	8.3	15	12.8	Ref.	
House							0.111**
Masonry	74	23	31.9	51	43.6	Ref.	
Others	115	49	68.1	66	56.4	1.64	

*OR - Odds Ratio; **P-value ≤ 0.20, variables included in the adjusted logistic regression model.

Regarding clinical parameters, the presence of polypharmacy was associated, as expected, with a greater number of chronic diseases (p<0.001) and inappropriate medication prescription (p<0.001), with worse gait and balance functionality (p = 0.019) and a higher rate of falls (p = 0.031) (Table 2).

**Table 2:** Univariate analysis of factors associated with polypharmacy in older adults followed by the Family Health Strategy according to health profile. Guarapuava, Paraná, Brazil, 2022

Variables	Total	Polypharmacy				OR*	p value
		Yes		No			
	n	n	%	n	%		
Chronic diseases							<0.001**
No	28	1	1.4	27	23.1	21.3	
Yes	161	71	98.6	90	76.9	Ref.	
Sleep classification							0.001**
Normal	102	28	38.9	74	63.2	Ref.	
Altered	87	44	61.1	43	36.8	2.70	
MMSE							0.079**
Normal	119	51	70.8	68	58.1	Ref.	
Altered	70	21	29.2	49	41.9	0.57	
GDS							0.010**
Normal	131	42	58.3	89	63.2	Ref.	
Altered	58	30	41.7	28	36.8	2.27	
Falls							0.031**
Yes	108	34	47.2	74	63.2	1.92	
No	81	38	52.8	43	36.8	Ref.	
ADL score							0.382
Normal	157	62	86.1	95	81.2	Ref.	
Altered	32	10	13.9	22	18.8	0.69	
Caregiver							0.658
Without	141	55	76.4	86	73.5	0.85	
With	48	17	23.6	31	26.5	Ref.	
Family Apgar							0.800
Highly functional	148	58	80.6	90	76.9	Ref.	
Moderately functional	19	6	8.3	13	11.1	0.98	
Severely dysfunctional	22	8	11.1	14	12.0	0.88	
Beers							< 0.001**
Yes	147	40	55.6	95	81.2	Ref.	
No	72	32	44.4	22	18.8	3.45	
Continuous variables		Mean	SD	Mean	SD		p value
POMA score		18.2	7.536	20.7	6.801	0.95	0.019**
MAN score		11.1	2.389	11.4	2.444	0.984	0.322

GDS, AVD, Beers, POMA, MAN; *OR - Odds Ratio; **P-value ≤ 0.20, variables included in the adjusted logistic regression model; SD – standard deviation.

In Table 3, multivariate analysis showed that the prevalence of polypharmacy increases by 2.69 times the chance of prescribing inappropriate medications (p=0.008), by 2.07 times the chance of worsening sleep quality (p=0.048), and was associated with blacks and browns (OR 2.22 – p=0.029).

**Table 3:** Multiple analysis of factors associated with polypharmacy in older adults. Guarapuava, Paraná, Brazil, 2022

Variables	OR* (IC95%)	p value
Beers	2.69 (1.29-5.62)	0.008
Altered sleep	2.07 (1.00-4.29)	0.048
Non-white race/color	2.22 (1.08-4.55)	0.029

*Model adjusted by GDS, gender and MMSE. *OR - Odds Ratio.

With regard to the investigation of associations between PIM and sociodemographic factors, the association with lower levels of education was found to be significant in the univariate analysis, with p = 0.041.

The investigation with clinical variables of global assessment of older adults, similar to what was found for polypharmacy, showed that worse sleep quality (p=0.021) and functionality index (p=0.039) were associated with PIM use among older adults. Additionally, PIM prescription and use was associated with the degree of family functionality (p<0.001), being more frequent among older adults without caregiver support (p=0.020) (Table 4).

**Table 4:** Univariate analysis of factors associated with Beers in older adults followed by the Family Health Strategy according to health profile. Guarapuava, Paraná, Brazil, 2022

Variables	Total	Beers		Non-Beers		OR*	p value
	n	n	%	n	%		
Chronic diseases							0.070**
No	28	4	7.4	24	14.8	2.70	
Yes	161	50	92.6	111	85.2	Ref.	
Sleep classification							0.021**
Normal	102	22	40.7	80	59.3	Ref.	
Altered	87	32	59.3	55	40.7	2.11	
MMSE							0.095**
Normal	119	39	72.2	80	59.3	Ref.	
Altered	70	15	27.8	55	40.7	0.55	
GDS							0.397
Normal	131	35	64.8	96	71.1	Ref.	
Altered	58	19	35.2	39	28.9	1.33	
Falls							0.114**
Sim	108	26	48.1	82	60.7	1.66	
No	81	28	51.9	53	39.3	Ref.	
ADL score							0.425
Normal	157	43	79.6	114	84.4	Ref.	
Altered	32	11	20.4	21	15.6	1.38	
Caregiver							0.020**
Without	141	34	47.2	107	91.5	2.24	
With	48	20	27.8	28	23.9	Ref.	
Family Apgar							<0.001**
Highly functional	148	32	59.3	116	85.9	Ref.	
Moderately functional	19	8	14.8	11	8.1	2.63	
Severely dysfunctional	22	14	25.9	8	5.9	6.34	
Polypharmacy							<0.001**
Yes	147	22	40.7	95	70.4	Ref.	
No	72	32	59.3	40	29.6	3.45	
Continuous variables		Mean	SD	Mean	SD		p value
POMA score		18.0	7.431	20.5	6.892	0.95	0.039**
Mini nutritional score		10.8	2.546	11.5	2.353	0.88	0.084**
Overall nutritional score		12.5	2.448	13.2	2.451	0.85	0.062**

*OR - Odds Ratio; **P-value ≤ 0.20, variables included in the adjusted logistic regression model; SD – standard deviation.

Multiple analysis showed that, regardless of the other variables, polypharmacy increases the chance of PIM by 3.92 times (p=0.001). Lack of caregiver support was associated with 3.74 times more chances of prescription and use of these medications (p=0.002), data corroborated by the association with the degree of functionality of families, since there were 13.79 times more chances of PIM in severely dysfunctional families (p<0.001) (Table 5).

**Table 5:** Multiple analysis of factors associated with Beers. Guarapuava, Paraná, Brazil, 2022

Variables	OR* (95%CI)	p value
Polypharmacy	3.92 (1.77-8.69)	0.001
Family Apgar		
Moderately functional	4.73 (1.50-14.92)	0.008
Severely dysfunctional	13.79 (4.07-46.75)	<0.001
Without caregiver	3.74 (1.61-8.70)	0.002
MMSE score	0.43 (0.18-0.98)	0.047

Model adjusted for chronic diseases; *OR - Odds Ratio.

Interestingly, altered cognitive function, in this study, was configured as a factor related to the reduction in PIM prescription among older adults (OR 0.43 – 95%CI 0.18 – 0.98) (Table 5).

## DISCUSSION

Care for older adults with hypertension in primary care services requires innovative actions, based on proper recognition of older adults so that they are effective. It is not possible to treat chronic illness without recognizing older adults’ specific needs. Thus, in the present study, with hypertension as a key condition for approaching older adults, the presence of polypharmacy and the rate of inappropriate medication prescription and use were assessed, considering that both phenomena, associated with hypertension, have unfavorable outcomes that must be prevented.

In the same way that hypertension is frequent among older adults, antihypertensive drug use represents the most used class of medication in this population^(14)^. In the present study, although most older adults were using antihypertensive monotherapy, 38.09% were using five or more medications simultaneously, a condition known as polypharmacy^(15)^.

Regardless of the health condition, the presence of polypharmacy increases the chances of hospital admission, falls and death among older adults^(8)^. The rate found was higher than that of a study carried out in the five Brazilian regions, which was 21% among hypertensive individuals^(16)^, and lower than that of the same study, when heart disease is taken as the basis, which was 42%. It should be noted that, however, in the group of older adults with hypertension assessed in the present study, other chronic diseases and cardiovascular disorders were also present. Therefore, our data are similar to the aforementioned study, strengthening the evidence on the extent of multiple medication prescription and use in this population.

Multidimensional assessment showed that polypharmacy was associated with inappropriate medication prescription, similar to other studies^(17,18)^, a fact that increases the risks of morbidity and mortality and disability among older adults. Both polypharmacy and PIM prescription are neglected factors in care for older adults, including in the context of hypertension, since continuous prescription antihypertensive drug use not indicated in this population was found.

Although there is content validation in Brazil for Beers and STOPP/START criteria for PIM for use in older adults^(19)^, there is no list of PIM considering the Brazilian pharmacopoeia. Unlike what is observed in other countries, such as France^(20)^, Austria^(21)^, Turkey^(22)^ and the United States^(23)^, consensuses are established for managing medications with potential risk for older adults, considering the drugs available in these countries. In hypertension management, it has already been established that using inappropriate antihypertensive drugs, such as those found in this study, associated with polypharmacy, increases the chances of falls, hospital admissions, frailty and deaths among older adults^(23)^.

The presence of polypharmacy was more frequent among black and brown people, and was associated with worse sleep quality. Few studies have investigated the effects of race and other sociodemographic aspects on polypharmacy, but, in the study by Assari and Bazargan^(24)^, race/ethnicity, age, marital status and employment did not correlate with polypharmacy. However, female, Low education and low income were associated with higher odds of polypharmacy among participants. As low income and less education are more frequent among non-whites in Brazil, the results of this study help to highlight the negative impact of social inequality on health status, with polypharmacy being yet another consequence that should be considered in this context.

Sleep disorders, such as fragmentation and daytime sleepiness, are frequent among older adults and are associated with worse health status^(16, 25)^. Antihypertensives, antidepressants, antiepileptics, corticosteroids, decongestants, caffeinated analgesics, and diuretics are among the many medications that have been linked to sleep disorders^(26, 27)^. The association found in this study between polypharmacy and worse sleep quality corroborates the results found in a study by Hamza, Saber and Hassan^(17)^ among hospitalized older adults and among older adults who underwent Garfinkel’s clinical protocol for drug prescription^(28)^. However, only the number of drugs in itself, apparently, does not establish evidence of the harms of this relationship, but the combination of prescribed drugs^(25)^.

Our results showed a high prevalence of PIM prescription to older adults, including antihypertensive classes. Moreover, the multivariate model demonstrated complex associations that can and should guide medication management for older adults in primary care. As already demonstrated, multiple drug prescription and use increase the chances of PIM^(29)^, however, in this study, this happens in scenarios of poor family support and absence of a caregiver, increasing the health risks of older adults. This data can be highlighted as an innovative result of the present study, by making explicit an aspect rarely discussed about PIM for older adults and polypharmacy.

As for the type of PIM, in the present study, proton pump inhibitor continuous use was the most prevalent (20.10%). A similar result was found in the study by Aires *et al*.^(30)^, carried out with more than 400 older adults. This fact demonstrates a peculiar Brazilian reality, since international, population-based studies demonstrate that antihistamines are the most used PIM^(31)^, some forms of insulin^(32)^, antihypertensive and cardiovascular medications^(33)^ and antidepressants^(8)^. It should be noted, at this point, that proton pump inhibitor use has been considered harmless, innocuous to older adults’ systemic health and with only symptomatic action^(34)^, thus denying the scientific evidence of the deleterious effects of this class of drugs, such as increased mortality rates^(35)^.

The advanced phenomenon of population aging exposes specificities of older adults that go beyond the number of diseases, such as the importance of support networks and caregivers, especially for the oldest old. Therefore, the results that showed in this study greater chances of PIM use among older adults without caregiver support, or in a dysfunctional family context, stand out in terms of their potential to guide more qualified care in these conditions of precarious support network.

Certainly, the greater number of diseases favors inappropriate medication prescription and increases older adults’ fragility, but this greater number of diseases must also be considered from the perspective of the need for support and the positive impacts of their strengthening, not only from the point of view of perspective of medicalization^(28)^. Older adults without support and with low education, as is the profile shown here, may have more difficulties in self-care and in understanding prescribed guidelines and treatments, which is why they are more subject to self-medication, use of wrong dosage, accumulation of prescriptions medical conditions and overuse of health services. Therefore, medication management for older adults who use Basic Health Units, especially in care programs aimed at hypertensive patients, must consider the aspects presented in the present study.

Another finding of this study was that cognitive dysfunction was characterized as a protective factor for PIM prescription and use among older adults with hypertension. It is interesting to note that the systematic review study corroborates that the prevalence of PIM was significantly lower in those with dementia or cognitive decline than among older adults without such disorders^(36)^. The condition of cognitive decline was configured as a factor that imposed greater attention on prescribers and, therefore, better drug therapy.

If the recognition of a factor, as a reason for greater attention to prescription, can qualify drug therapy, it is noteworthy that the results of this study can also qualify multidimensional care for older adults in primary care services, especially in the context of hypertension, which is the central focus of the search and care for this population in these services.

### Study limitations

One of the limitations of this study is the in-depth exploration of a single location and, therefore, the results do not allow for large-scale extrapolations.

### Contributions to nursing, health or public policy

Similarities with studies with a larger sample size and the identification of variables little explored in the literature, associated with polypharmacy practice and PIM prescription and use among older adults with hypertension, subsidize the highlighted results and their potential both for nurses’ clinical and management practice and for the review of strategic actions and drug prescription in the area of primary care.

## CONCLUSIONS

This research identified a prevalence of 39.09% of polypharmacy and 28.57% of PIM use, in addition to associated factors that are useful to compose a clinical profile of hypertensive older adults in the context of primary care. There was a significant association between polypharmacy and PIM use, altered sleep and race. PIM use, on the other hand, was associated with polypharmacy, worse family functioning and the absence of a caregiver, regardless of the other variables analyzed. It is concluded that the prevalence of polypharmacy and PIM use among older adults with hypertension is high, especially among those with a more vulnerable social profile, and factors are a priority for qualified care from primary care teams.
